# Cumulative inflammatory burden is independently associated with increased arterial stiffness in patients with psoriatic arthritis: a prospective study

**DOI:** 10.1186/s13075-015-0570-0

**Published:** 2015-03-17

**Authors:** Jiayun Shen, Qing Shang, Edmund K Li, Ying-Ying Leung, Emily W Kun, Lai-Wa Kwok, Martin Li, Tena K Li, Tracy Y Zhu, Cheuk-Man Yu, Lai-Shan Tam

**Affiliations:** Department of Medicine & Therapeutics, The Prince of Wales Hospital, The Chinese University of Hong Kong, Shatin, Hong Kong China; Department of Rheumatology and Immunology, Singapore General Hospital, Outram Road, Singapore 169608, Singapore, Singapore; Department of Medicine and Geriatrics, Taipo Hospital, Taipo, Hong Kong China

## Abstract

**Introduction:**

The aim of this study was to examine whether the cumulative inflammatory burden is associated with an increase in arterial stiffness in a prospective cohort of psoriatic arthritis (PsA) patients.

**Methods:**

In total, 72 PsA patients were followed for a median of 6.5 years. Cumulative inflammatory burden was represented by the cumulative averages of repeated measures of erythrocyte sedimentation rate (ca-ESR) and C-reactive protein (ca-CRP). Brachial-ankle pulse wave velocity (PWV) was measured at the last visit. We also included 47 healthy controls for PWV assessment.

**Results:**

PWV was significantly higher in PsA patients compared with healthy controls after adjustment for age, gender and body weight (1466 ± 29 cm/s versus 1323 ± 38 cm/s, *P* = 0.008). PsA patients were divided into two groups based on whether their PWV value is ≥1450 cm/s (High PWV group, N = 38) or <1450 cm/s (Low PWV group, N = 34). The High PWV group had a significantly higher ca-ESR (29 (19 to 44) versus 18 (10 to 32) mm/1st hour, *P* = 0.005) and ca-CRP (0.7 (0.3 to 1.4) versus 0.4 (0.2 to 0.7) mg/dl, *P* = 0.029). Using regression analysis, high ca-ESR (defined as ≥75th percentile: 37 mm/1st hour) was associated with a higher likelihood of being in the High PWV group (odds ratio (OR): 9.455 (1.939 to 46.093), *P* = 0.005, adjusted for baseline clinical and cardiovascular risk factors; and 9.111 (1.875 to 44.275), *P* = 0.006, adjusted for last visit parameters).

**Conclusions:**

Cumulative inflammatory burden, as reflected by ca-ESR, was associated with increased arterial stiffness in PsA patients even after adjustment for cardiovascular risk factors, emphasizing the important role of chronic inflammation in accelerating the development of cardiovascular risks in PsA patients.

## Introduction

Psoriatic arthritis (PsA) is a chronic inflammatory arthritis associated with an increased risk of subclinical [1,2], clinical cardiovascular disease (CVD) [3] and early cardiovascular (CV) mortality [4]. Chronic inflammation plays a pivotal role in the pathogenesis of subclinical CVD in PsA patients [5,6]. Inflammation is involved in foam cell formation, endothelial dysfunction and Th1 cytokine production, which leads to the development of arterial dysfunction [7] and atherosclerotic plaque [8]. However, inflammatory markers including erythrocyte sedimentation rate (ESR) or C-reactive protein (CRP) failed to differentiate PsA patients with or without subclinical atherosclerosis in cross-sectional studies [1,9], most likely because PsA is a chronic relapsing condition in which inflammation may fluctuate over time. The chronic inflammatory burden may be better represented by the cumulative average of inflammatory markers than the measurement of these markers at a single time point. Eder and colleagues have recently reported that increased inflammatory burden over time, as reflected by cumulative average of ESR, is associated with the extent of atherosclerotic plaques in PsA patients from a prospective cohort [10]. This association is attenuated after adjustment for traditional CV risk factors. In patients with rheumatoid arthritis (RA), a prospectively study also demonstrated that higher average CRP levels are associated with incident or progressive plaque, but only in patients with high CVD risk [11]. In RA, the average CRP correlates with the presence of subclinical atherosclerosis measured by carotid intima-media thickness (IMT) [12]. Also, the average CRP is associated with an increased risk of CV events and mortality in patients with long-standing RA [13]. Whether chronic inflammation can accelerate atherogenesis independently or mediate it via adverse modification of CV risk factors remains uncertain.

Arterial stiffness is an independent predictor of CV events and mortality [14]. Pulse wave velocity (PWV) is a measure of early structural vascular changes, which is determined by the elasticity and other properties of the artery, and is correlated with arterial distensibility and stiffness. An increase in brachial-ankle PWV by 100 cm/s corresponds to an age-, sex-, and risk factor-adjusted increase of 12% in total CV events, and 13% in CV mortality, respectively [15]. By applying a cutoff value of 1450 cm/s, brachial-ankle PWV was found to discriminate normal subjects from those with CVD or atherosclerotic risk factors with sensitivity of 62.1% and specificity of 69.5% in a large-scale population-based study involving 21,094 Chinese subjects [16]. Two previous case–control studies failed to find a correlation between ESR or CRP and PWV in PsA patients [17,18]. However, these studies were limited by small sample size and cross-sectional study design and were unable to assess the effect of cumulative inflammation over time. The effect of cumulative inflammatory burden in arterial stiffness in patients with RA is controversial [19,20]. Whether cumulative inflammatory burden over time may affect arterial stiffness as assessed by PWV in PsA has never been explored. We hypothesize that PsA patients with higher cumulative inflammatory burden (as measured by cumulative averages of the area under the curve (AUC) for ESR and CRP) would have increased arterial stiffness. In this study, we first compared the arterial stiffness between PsA patients and healthy controls. Second, we examined whether the cumulative inflammatory burden over time is associated with an increase in arterial stiffness in a prospective cohort of PsA patients.

## Methods

### Patients and healthy controls

Eighty-two PsA patients who participated in a prior subclinical study of atherosclerosis in PsA [1] were recruited for a PWV assessment between 2012 and 2013. Briefly, all participants fulfilled the Classification of Psoriatic Arthritis (CASPAR) criteria [21] and had been prospectively followed at the rheumatology clinic of two regional hospitals (The Prince of Wales Hospital and the Alice Ho Miu Ling Nethersole Hospital) since 2006 to 2007 (baseline visit). Exclusion criteria at baseline included overt CVD (including myocardial infarction, angina, stroke, and transient ischemic attack), inability to provide informed consent, clinically significant renal disease (serum creatinine level >270 mol/L), or pregnancy. Patients were assessed by rheumatologists every 4 to 6 months, which included a complete history, physical examination and laboratory evaluation: 72 patients who completed the last follow-up visit and had a successful brachial-ankle PWV assessment were included in the analysis.

We recruited 47 healthy controls from a broad spectrum of hospital staff. None of the controls had a known history of hypertension, diabetes, hyperlipidemia, or overt CVD (including myocardial infarction, angina, stroke, or transient ischemic attack), or family history of CVD.

Ethics approval was obtained from the Ethics Committee of The Chinese University of Hong Kong-New Territories East Cluster Hospitals, and written informed consent was obtained from all participants according to the Declaration of Helsinki.

### Clinical interview at baseline and last follow-up visit

Pain and physicians’ and patients’ global assessments were evaluated using a 100-point visual analog scale, where 0 indicated excellent well-being and 100 indicated feeling extremely unwell. Physical examination included recording the number of tender and swollen joints using the 68 tender-joint/66 swollen-joint count, the presence of dactylitis, and the number of permanently deformed joints. The Health Assessment Questionnaire (HAQ) was used to evaluate physical function [22], and the Psoriasis Area and Severity Index (PASI) was used to assess the extent of skin involvement [23]. Overall disease activity was assessed using the Disease Activity in Psoriatic Arthritis (DAPSA) and Minimal Disease Activity (MDA) scores [24]. Anthropomorphic measurements including height, weight, and waist and hip circumference, and two consecutive blood pressure (BP) readings in the sitting position and heart rate were recorded. Other data obtained from PsA patients through the interview and chart review included smoking and drinking habits, history of diabetes, hypertension, hypercholesterolemia and overt CVD. Drug history was retrieved from case notes or elicited during the clinical assessment. All patients were interviewed and examined using standardized data collection instruments.

### Laboratory tests

Complete blood count, liver and renal function tests, ESR and CRP were checked at every visit. Fasting blood glucose, and lipid profile (total cholesterol (TC), low-density lipoprotein-cholesterol (LDL), high-density lipoprotein-cholesterol (HDL), and triglycerides) were checked at baseline and the last visit. Cumulative inflammation over time was represented by the cumulative averages of ESR (ca-ESR) and CRP (ca-CRP).

### Pulse wave velocity

Brachial-ankle PWV was assessed noninvasively in subjects in the supine position by a dedicated tonometry system (Non-Invasive Vascular Profile Device VP-2000; Omron Healthcare, Inc, Bannockburn, IL, USA) as described previously [25]. All PWV measurements were performed twice at each side of the body by a single skilled operator. The means of overall PWV measurements were recorded. The intra-class correlation coefficient (ICC) for intraobserver reliability was 0.84 [26].

### Statistical analysis

Results are expressed as mean ± SD or median (interquartile range) as appropriate. Comparisons between two groups were assessed using the Student’s *t*-test or Mann–Whitney *U*-test for continuous variables and the chi-square (χ^2^) test for categorical variables. Analysis of covariance (ANCOVA) was used to compare means of PWV between PsA and control subjects by adjusting for parameters that were distributed differently between groups. Spearman’s correlation was used to evaluate bivariate correlation. ca-ESR and ca-CRP were calculated from the AUC of all available measurements divided by the total number of months of follow-up. Univariate analysis was performed to ascertain the association between clinical parameters and PWV. The cutoff value for CV and atherosclerotic risk, which was derived from the large-scale Chinese population-based study [16], was used to divide the PsA patients into a high-PWV group (≥1,450 cm/s) or low-PWV group (<1,450 cm/s). Multivariable logistic regression analysis was used to determine the independent predictor(s) for being in the high-PWV group. All variables with *P* <0.1 in the univariate analysis were included in the multivariate analysis. All statistical analyses were conducted using IBM SPSS Statistics Version 22 (IBM, Armonk, NY, USA). A minimal level of significance of *P* <0.05 is used.

## Results

### Clinical features of PsA patients

A total of 72 (36 male and 36 female) PsA patients were included in the analysis. At baseline, the mean ± SD age was 49.6 ± 11.7 years and the median (IQR) disease duration was 9.2 (2.4 to 14.1) years. The median follow-up duration from baseline to the time of PWV assessment (last visit) was 6.5 (range: 4.8 to 7.7) years. Table 1 summarized the clinical features of the patients at baseline and last visit. Compared with baseline, significant improvement in disease activity parameters (number of tender and swollen joint counts, pain, DAPSA and CRP) and physical function (HAQ) were observed at the last visit, although the damaged joint count increased. CV risk factors remained stable except for systolic blood pressure (SBP) and HDL levels decreased. More patients were taking anti-hypertensive drugs, statins and biologic disease modifying anti-rheumatic drugs (DMARDs) at the last visit.

**Table 1:** **Clinical features at baseline and last visit in all PsA patients**

	**Baseline**	**Last visit**	***P*** **-value**
**Male gender, n (%)**	36 (50.0%)		
**Age, years**	49.6 ± 11.7	55.9 ± 11.6	
**Psoriatic arthritis (PsA) characteristics**			
PsA disease duration, years	9.2 (2.4 to 14.10)	15.7 (8.5 to 21.2)	
Tender joint count, 0 to 68	2 (0 to 8)	1 (0 to 4)	0.033
Swollen joint count, 0 to 66	0 (0 to 3)	0 (0 to 1)	0.032
Damaged joint count, 0 to 68	2 (0 to 5)	2 (0 to 6)	0.031
Visual analog scale pain, 0 to 100	50 (30 to 66)	30 (20 to 58)	0.001
Patients’ global assessment, 0 to 100	50 (30 to 60)	40 (20 to 60)	0.298
Physicians’ global assessment, 0 to 100	20 (0 to 30)	20 (5 to 35)	0.683
Psoriasis Area and Severity Index, 0 to 72	2.6 (0.9 to 7.5)	1.8 (0.4 to 6.3)	0.309
Health assessment questionnaire, 0 to 3	0.38 (0.12 to 0.94)	0.25 (0 to 0.69)	0.043
Minimal disease activity, n (%)	16 (22.2%)	14 (19.4%)	0.539
Disease Activity in Psoriatic Arthritis, 0 to 164	16 (8 to 21)	11 (6 to 16)	0.010
Erythrocyte sedimentation rate, mm/1st h	23 (10 to 37)	18 (8 to 34)	0.357
C-reactive protein, mg/dl	0.4 (0.2 to 1.3)	0.3 (0.1 to 0.7)	0.016
**Cardiovascular risk factors**			
Body weight, kg	65.2 ± 12.0	65.7 ± 11.6	0.436
Body height, cm	161 ± 8		
Systolic blood pressure, mmHg	136 ± 22	127 ± 15	<0.001
Hypertension, n (%)	36 (50.0%)	44 (61.1%)	0.555
Diabetes, n (%)	16 (44.4%)	16 (44.4%)	1.000
Framingham 10-year CVD risk >10%, n (%)	30 (41.7%)	36 (50.0%)	0.316
Total cholesterol, mmol/L	5.1 ± 0.9	4.9 ± 0.8	0.073
High-density lipoprotein cholesterol, mmol/L	1.6 ± 0.5	1.4 ± 0.4	<0.001
Triglycerides, mmol/L	1.5 ± 0.9	1.5 ± 0.9	0.558
Fasting glucose, mmol/L	5.5 ± 1.3	5.5 ± 1.6	0.861
**Medications, n (%)**			
Anti-hypertensive drugs	20 (27.8%)	42 (58.3%)	<0.001
Statins	1 (1.4%)	14 (19.4%)	<0.001
Nonsteroidal anti-inflammatory drugs	33 (45.8%)	31 (43.1%)	0.737
Steroids	4 (5.6%)	3 (4.2%)	0.999
Disease-modifying antirheumatic drugs	36 (50.0%)	40 (55.6%)	0.504
Biologics	0 (0%)	11 (15.4%)	0.001

Values are presented as number (percentage), median (interquatile range) or mean ± SD. CVD, cardiovascular disease.

### PWV in PsA patients and control subjects

**Figure 1:**
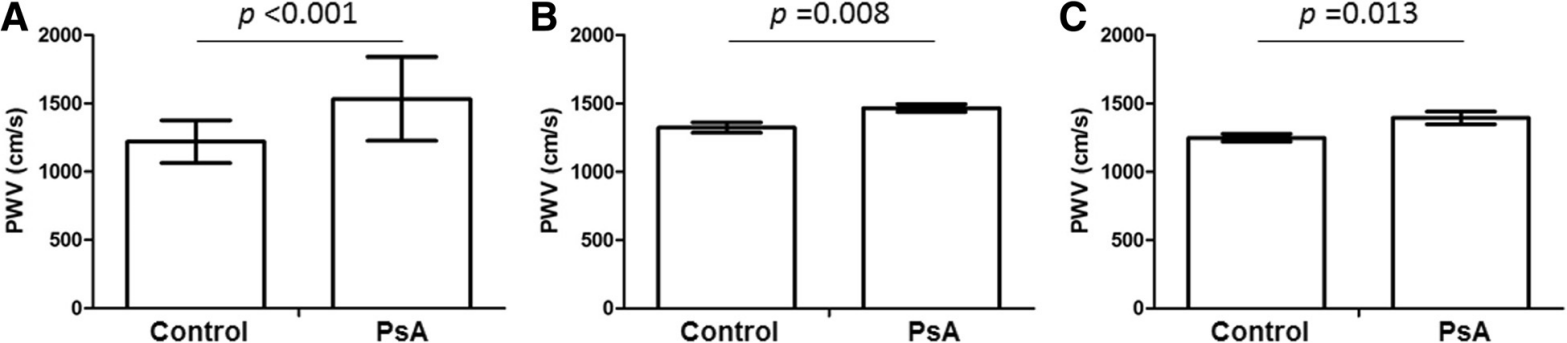
**Pulse wave velocity (PWV) in control subjects and patients with psoriatic arthritis (PsA). (A)** Unadjusted mean in control subjects (n = 47) and all PsA patients (n = 72). **(B)** Mean adjusted by age, gender and body weight in control subjects and all PsA patients. **(C)** Mean adjusted by age, gender and body weight in control subjects and PsA patients without hypertension, diabetes or hyperlipidemia (n = 20).

The mean PWV in PsA patients and control subjects were 1,533 ± 307 cm/s and 1,219 ± 157 cm/s, respectively (*P* <0.001) (Figure 1A). The control subjects were significantly younger (43.1 ± 10.2 years versus 55.9 ± 11.6 years, *P* <0.001), had higher proportion of women (72.3% versus 50.0%, *P* = 0.015) and a lower body weight (58.2 ± 8.7 kg versus 65.7 ± 11.6 kg, *P* <0.001) at PWV assessment compared with the PsA patients. However, after adjustment for age, gender and body weight, the adjusted mean PWV was still significantly greater in PsA patients compared with control subjects (1,466 ± 29 cm/s versus 1,323 ± 38 cm/s, *P* = 0.008) (Figure 1B). If patients with hypertension, diabetes or hyperlipidemia were excluded, the age, gender, and body weight-adjusted mean for PsA patients (n = 20) and control subjects were 1,394 ± 46 and 1,248 ± 29 cm/s, respectively (*P* = 0.013) (Figure 1C).

### Association between traditional cardiovascular risk factors and PWV

The associations between PWV and clinical features at both baseline and the last visit (PWV assessment visit) are shown in Table 2. At PWV assessment, patients in the high-PWV group (n = 38) were significantly older, had higher SBP and Framingham 10-year CVD risk score, were more likely to have diabetes and hypertension, and to be treated with anti-hypertensive drugs compared with the low-PWV group (n = 34) (all *P* <0.05).

**Table 2:** **Clinical features at baseline and last follow-up visit in patients in the high and low pulse-wave velocity (PWV) groups**

	**Baseline**			**Last visit**		
	**Low PWV**	**High PWV**	***P*** **-value**	**Low PWV**	**High PWV**	***P*** **-value**
**Male gender, n (%)**	17 (50.0%)	19 (50.0%)	1.000	17 (50.0%)	19 (50.0%)	1.000
**Age, years**	**43.3 ± 10.2**	**55.2 ± 10.0**	**<0.001**	**49.6 ± 10.1**	**61.6 ± 9.9**	**<0.001**
**Psoriatic arthritis (PsA) characteristics**						
PsA disease duration, years	**7.6 ± 7.0**	**10.7 ± 7.5**	**0.073**	**13.9 ± 7.2**	**17.1 ± 7.8**	**0.075**
Tender joint count, 0 to 68	2 (0 to 6)	3 (0 to 8)	0.551	1 (0 to 3)	1 (0 to 5)	0.248
Swollen joint count, 0 to 66	0 (0 to 2)	1 (0 to 3)	0.827	0 (0 to 1)	0 (0 to 1)	0.376
Damaged joint count, 0 to 68	1 (0 to 3)	2 (0 to 6)	0.118	**0 (0 to 4)**	**4 (0 to 8)**	**0.010**
Visual analog scale pain, 0 to 100	40 (20 to 60)	50 (30 to 70)	0.212	30 (20 to 50)	30 (20 to 60)	0.964
Patients’ global assessment, 0 to 100	50 (20 to 60)	50 (40 to 60)	0.258	50 (20 to 60)	33 (20 to 60)	0.654
Physicians’ global assessment, 0 to 100	10 (0 to 20)	20 (5 to 30)	0.462	19 (6 to 35)	20 (7 to 30)	0.399
Psoriasis Area and Severity Index, 0 to 72	2.6 (1.0 to 7.3)	3.0 (0.7 to 7.7)	0.879	1.8 (0.6 to 5.4)	1.7 (0.3 to 7.4)	0.879
Health assessment questionnaire, 0 to 3	0.25 (0.13 to 0.63)	0.56 (0.13 to 1.00)	0.120	0.13 (0 to 0.50)	0.38 (0 to 1.13)	0.146
Minimal disease activity, n (%)	10 (29.4%)	6 (15.8%)	0.165	6 (17.6%)	8 (21.1%)	0.715
Disease Activity in Psoriatic Arthritis, 0 to 164	14 (7 to 19)	17 (12 to 22)	0.132	11 (7 to 15)	12 (5 to 18)	0.827
Erythrocyte sedimentation rate, mm/1st h	**16 (7 to 34)**	**27 (13 to 56)**	**0.048**	**11 (5 to 22)**	**29 (13 to 56)**	**<0.001**
C-reactive protein, mg/dl	0.3 (0.1 to 1.2)	0.5 (0.2 to 1.3)	0.527	**0.2 (0.1 to 0.5)**	**0.4 (0.2 to 0.8)**	**0.023**
**Cardiovascular risk factors**						
Body weight, kg	66.1 ± 12.4	64.4 ± 11.8	0.540	**68.1 ± 12.0**	**63.6 ± 11.0**	**0.099**
Body height, cm	161 ± 8	161 ± 9	0.891			
Systolic blood pressure, mmHg	132 ± 23	140 ± 21	0.113	**121 ± 13**	**132 ± 16**	**0.002**
Hypertension, n (%)	**10 (29.4%)**	**26 (68.4%)**	**0.001**	**15 (44.1%)**	**29 (76.3%)**	**0.005**
Diabetes, n (%)	**3 (8.8%)**	**13 (34.2%)**	**0.010**	**3 (8.8%)**	**13 (34.2%)**	**0.010**
Framingham 10-year CVD risk >10%, n (%)	**8 (23.5%)**	**22 (57.9%)**	**0.003**	**9 (26.5%)**	**27 (71.1%)**	**<0.001**
Total cholesterol, mmol/L	5.1 ± 0.9	5.1 ± 1.0	0.814	5.0 ± 1.0	4.8 ± 0.7	0.468
High-density lipoprotein cholesterol, mmol/L	1.6 ± 0.5	1.6 ± 0.5	0.782	1.5 ± 0.4	1.4 ± 0.4	0.467
Triglycerides, mmol/L	1.4 ± 0.7	1.6 ± 1.1	0.285	1.3 ± 0.8	1.5 ± 1.0	0.363
Fasting glucose, mmol/L	5.3 ± 1.6	5.7 ± 1.0	0.227	5.2 ± 1.0	5.6 ± 1.9	0.287
**Medications, n (%)**						
Anti-hypertensive drugs	**3 (8.8%)**	**17 (44.7%)**	**<0.001**	**14 (41.2%)**	**28 (73.7%)**	**0.005**
Statins	0 (0%)	1 (2.6%)	1.000	7 (20.6%)	7 (18.4%)	0.817
Nonsteroidal anti-inflammatory drugs	13 (38.2%)	20 (52.6%)	0.221	16 (47.1%)	15 (39.5%)	0.516
Steroids	2 (5.9%)	2 (5.3%)	0.999	1 (2.9%)	2 (5.3%)	0.999
Disease-modifying antirheumatic drugs	15 (44.1%)	21 (55.3%)	0.345	18 (52.9%)	22 (57.9%)	0.673
Biologics	0 (0%)	0 (0%)	N.S.	7 (20.6%)	4 (10.5%)	0.236

Variables with *P*-values <0.1 (values in bold text) were candidates for multivariate analysis. Values are presented as number (percentage), median (interquatile range), or mean ± SD.

### Association between disease-related parameters and PWV

Disease-related variables were compared between the two PWV groups (Table 2). The high-PWV group had a trend of longer disease duration (*P* <0.1). At baseline, the high-PWV group had significantly higher ESR levels compared with patients in the low-PWV group (*P* = 0.048). At PWV assessment, the high-PWV group had significantly higher damaged joint count, ESR and CRP levels.

### Association between cumulative inflammatory burden and PWV

**Figure 2:**
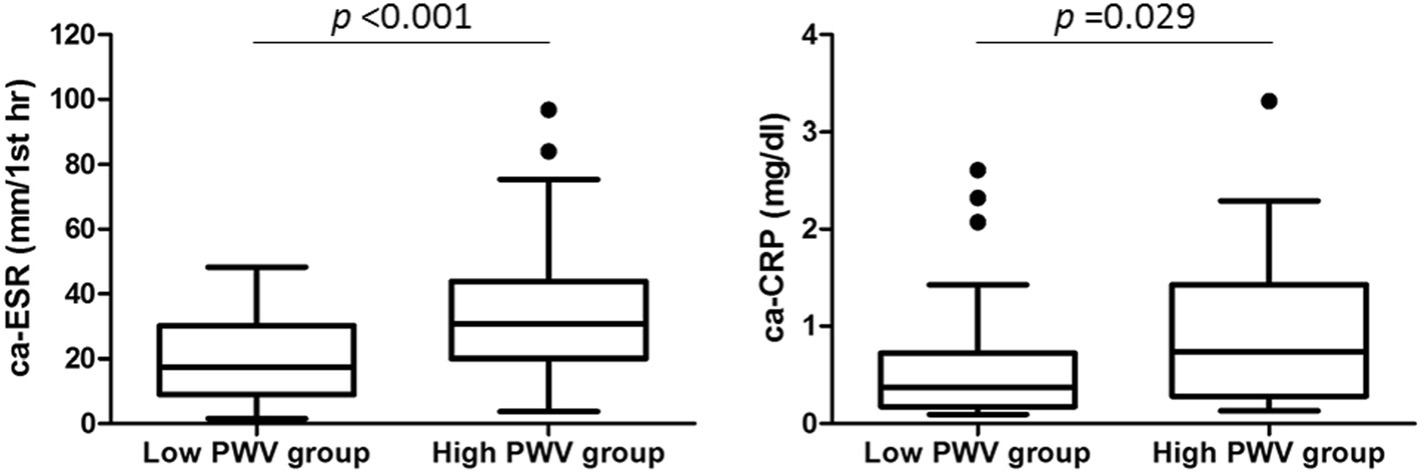
**Cumulative inflammatory burden and pulse wave velocity (PWV).** Cumulative average of erythrocyte sedimentation rate (ca-ESR) and C-reactive protein (CRP) in the low PWV group (PWV <1,450 cm/s, n = 34) and the high-PWV group (PWV ≥1,450 cm/s, n = 38).

The median ca-ESR was 24 (12 to 38) mm/1st h and the median ca-CRP was 0.6 (0.2 to 1.1) mg/dl in the PsA patients. There was a significant correlation between PWV and ca-ESR (Spearman’s rho 0.390, *P* = 0.001) but not ca-CRP (0.222, *P* = 0.061). The high-PWV group had significantly higher ca-ESR (31 (21 to 44) versus 17 (10 to 30) mm/1st h, *P* <0.001) and ca-CRP (0.7 (0.3 to 1.4) versus 0.4 (0.2 to 0.7) mg/dl, *P* = 0.029) compared with the low-PWV group (Figure 2).

The association between the cumulative inflammatory burden and PWV was assessed by regression analysis (Table 3). High ca-ESR (defined as ≥75th percentile: 37 mm/1st h) was associated with a higher likelihood of being in the high-PWV group after adjustment for other clinical and CV risk factors at baseline (OR 9.455 (95% CI 1.939, 46.093), *P* = 0.005) or last visit (OR 9.111 (95% CI 1.875, 44.275), *P* = 0.006) (Table 3). In contrast, high ca-CRP (defined as ≥75th percentile 1.2 mg/dl) was not associated with a higher likelihood of being in the high-PWV group (Table 3).

**Table 3:** **Association between cumulative inflammatory burden and high-PWV group by multivariable logistic regression**

**Model** ^**a**^	**Factors**	**Odds ratio**	**95% CI**	***P*** **-value**
Baseline^b^	High cumulative ESR^d^	9.455	1.939, 46.093	0.005
	High cumulative CRP^e^	1.736	0.294, 10.268	0.543
Last visit^c^	High cumulative ESR^d^	9.111	1.875, 44.275	0.006
	High cumulative CRP^e^	0.888	0.088, 9.007	0.920

^a^Adjusted for parameters at baseline or last follow up. ^b^Parameters entered: age, psoriatic arthritis (PsA) duration, hypertension, diabetes, Framingham risk score, use of anti-hypertension drugs, high erythrocyte sedimentation rate (ESR)^d^, cumulative average (ca)-ESR^d^ and ca-C-reactive protein (CRP)^e^. ^c^Parameters entered: age, PsA duration, body weight, systolic blood pressure, hypertension, diabetes, damaged joints count, use of anti-hypertension drugs, Framingham risk score, high ESR^d^ and CRP^e^, high ca-ESR^d^ and ca-CRP^e^. ^d^Defined as ≥75th percentile: 37 mm/1st h. ^e^Defined as ≥75th percentile: 1.2 mg/dl.

Older age was the other independent risk factor associated with a higher likelihood of being in the high-PWV group (adjusted for baseline parameters: OR 1.137 (95% CI 1.058, 1.223), *P* <0.001; adjusted for last-visit parameters: OR 1.135 (95% CI 1.056, 1.219), *P* = 0.001).

## Discussion

This is the first study to assess the association between cumulative inflammatory burden and arterial stiffness in PsA patients. High cumulative inflammatory burden as reflected by the ca-ESR was a predictor for higher PWV independently of traditional CV risk factors and other disease activity parameters. We also confirmed that PWV is increased in PsA patients compared with healthy control subjects in a larger cohort.

We have demonstrated a significant correlation between ca-ESR and PWV (*P* = 0.001) and a marginally significant correlation between ca-CRP and PWV (*P* = 0.061), suggesting that chronic inflammation may have a causative role in the development of arterial dysfunction in PsA patients. These results were consistent with previous findings that cumulative inflammatory burden (ca-ESR) was associated with increased aortic augmentation index in RA [19] and severity of carotid plaque in PsA [10]. One previous study reported that PWV associated with current CRP levels, but not with historical measures of cumulative ESR inflammatory burden in RA [20]. However, this study excluded patients with hypercholesterolemia and hypertension, and current smokers. In the current study ca-ESR, ca-CRP and single measurements of ESR and CRP at PWV assessment were associated with high PWV in the univariate analysis. Nonetheless, only ca-ESR was independently associated with high PWV in multivariate analysis after adjusting for other traditional CV risk factors and disease-related parameters, indicating that the cumulative inflammatory burden may better explain increased arterial stiffness than transient inflammatory status.

Different from ca-ESR, ca-CRP was not associated with PWV in multiple regression. A previous study from Eder *et al*. reported no association between ca-CRP and atherosclerosis in patients with PsA [10] while Giles *et al*. [11] reported that higher ca-CRP levels were associated with incident or progressive plaque, primarily in patients with elevated CVD risk in patients with RA. These seemingly contradictory findings may be explained by the relatively low levels of chronic inflammation that are commonly found in PsA compared with RA. A high-sensitivity (hs) CRP assay may be more informative, but was not available in our study. Serial monitoring of inflammatory markers including hsCRP should be considered in PsA patients.

Damaged joint count at the last visit was also associated with increased PWV in the univariate analysis. Indeed, damaged joint count could also represent the cumulative inflammatory burden and was found to be independently associated with atherosclerosis in RA [27]. However, the significance of damaged joint count was lost after adjusting for ca-ESR and other CV risk factors in PsA patients.

We also confirmed that arterial stiffness is increased in PsA patients. PWV was significantly higher in PsA patients after adjustment for age, gender and body weight, and even after PsA patients with hypertension, diabetes or hyperlipidemia were excluded. This is consistent with other studies with smaller case numbers that measured arterial stiffness by aortic PWV [17,18]. Costa *et al*. [17] reported increased PWV in 20 PsA patients compared with 20 controls. Soy *et al*. [18] also showed that PWV was higher in 9 PsA patients compared with 39 controls. In a previous study involving 73 PsA patients and 50 healthy controls, a significant increase in augmentation index was noticed, indicating impairment of both macrovascular and microvascular functions [28].

Although increased overall mortality in PsA patients has been reported in some [29-32] but not all [33,34] studies, previous data have consistently indicated an increased susceptibility to CVD and related mortality in PsA patients [3,35]. Our results indicated that the increased CVD and related mortalities may be partly mediated by increased arterial stiffness through persistent chronic inflammation. Inflammation accelerates subclinical atherosclerosis probably through adverse modification of the traditional CV risk factors [10]. In contrast, the association between cumulative inflammation and arterial stiffness was independent of traditional CV risk factors such as age, hypertension and diabetes. This may suggest that inflammation-induced arterial dysfunction is probably an early phase in the development of atherogenesis, and hence, PWV is a more sensitive marker reflecting changes in predominantly macrovascular functions in patients with rheumatic disease and chronic inflammation, compared to arterial remodeling as reflected by increased IMT and plaques.

PsA is associated with reduced levels of endothelial progenitor cells (EPCs) and impaired EPC function, leading to decreased release of nitric oxide (NO) [36]. Inflammatory cells such as macrophages and polymorphonuclear neutrophils produce a variety of matrix metalloproteinases (MMPs), which can alter the balance of elastin/collagen [37]. Chronic inflammation may also induce oxidative stress [7]. All these changes will lead to arterial stiffening. Inflammation also interacts with other important pathways such as advanced glycation end products (AGEs), which can irreversibly bind to collagens, resulting in stiffer AGE-linked collagen [38,39]. Moreover, AGEs can also promote inflammatory response through binding to the receptor (RAGE) and subsequently increase arterial stiffness [40,41]. TNF-α is a key cytokine involved in the pathogenesis of PsA [42], which can induce neutrophil chemotaxis, macrophage activation and superoxide production. This results in endothelial inflammation and dysfunction, and may contribute to the development of arterial damage [43]. Data from a non-randomized case–control study in patients with inflammatory arthritis (RA, PsA and ankylosing spondylitis) showed that long-term use of anti-TNF-α therapy may result in a significant improvement in PWV compared to the non-treated group [44]. Whether effective suppression of inflammation can improve arterial stiffness in PsA should be explored in future clinical trials.

The strength of our study was the inclusion of a large prospective cohort with long-term follow up. Patients with CV risk factors were not excluded so that our results can be generalized to the usual PsA patient population. Our study also has a few limitations. First, our results may not be applicable to PsA patients from other ethnic backgrounds. Second, the outcome of this study (PWV) is only a surrogate of clinical CV events. In patients with PsA or RA, there are virtually no data to suggest whether PWV is a good surrogate of future CVD events. Third, arterial stiffness was assessed by brachial-ankle PWV but not the gold standard, carotid-femoral PWV. However, brachial-ankle PWV is highly correlated with carotid-femoral PWV and may provide qualitatively similar information [45]. Fourth, only baseline and last-visit clinical and traditional risk factors were adjusted for. With regards to the association between cumulative inflammatory burden and arterial stiffness, we have only addressed the role of cumulative averages of ESR and CRP. Unfortunately, we did not have data on other disease activity measures, for example, DAPSA and MDA during all the visits. It would be of interest for a future study to include a measure such as average mean DAPSA, or to determine whether achieving MDA for a prolonged period of time could be a potential predictor of arterial stiffness. Last but not least, baseline PWV measurement was not available to assess the relationship between cumulative inflammatory burden or the effect of treatment and the changes in arterial stiffness. Further prospective studies should be conducted to address this issue.

## Conclusions

In conclusion, PsA patients have increased arterial stiffness compared with healthy control subjects. Cumulative inflammatory burden contributes to the increased arterial stiffness independent of traditional CV risk factors, suggesting that increasing arterial stiffness may be one of the mechanisms linking inflammation and CVD in PsA.

## Abbreviations

AGEs: Advanced glycation end products
ANCOVA: analysis of covariance
AUC: area under the curve
BP: blood pressure
ca-CRP: cumulative averages of CRP
ca-ESR: cumulative averages of ESR
CASPAR: Classification of Psoriatic Arthritis
CRP: C-reactive protein
CV: cardiovascular
CVD: cardiovascular disease
DAPSA: Disease Activity in Psoriatic Arthritis
EPCs: endothelial progenitor cells
ESR: erythrocyte sedimentation rate
HAQ: Health Assessment Questionnaire
HDL: high-density lipoprotein cholesterol
Hs: high-sensitivity
ICC: intra-class correlation coefficient
IMT: intima-media thickness
LDL: low-density lipoprotein cholesterol
MDA: minimal disease activity
MMP: matrix metalloproteinase
NO: nitric oxide
PASI: Psoriasis Area and Severity Index
PsA: psoriatic arthritis
PWV: pulse wave velocity
RA: rheumatoid arthritis
RAGE: Receptor of AGEs
SBP: systolic blood pressure
TC: total cholesterol

## References

[1] Tam LS, Shang Q, Li EK, Tomlinson B, Chu TT, Li M (2008). Subclinical carotid atherosclerosis in patients with psoriatic arthritis. Arthritis Rheum.

[2] Zhu TY, Li EK, Tam LS (2012). Cardiovascular risk in patients with psoriatic arthritis. Int J Rheumatol.

[3] Han C, Robinson DW, Hackett MV, Paramore LC, Fraeman KH, Bala MV (2006). Cardiovascular disease and risk factors in patients with rheumatoid arthritis, psoriatic arthritis, and ankylosing spondylitis. J Rheumatol.

[4] Gladman DD, Farewell VT, Wong K, Husted J (1998). Mortality studies in psoriatic arthritis: results from a single outpatient center. II. Prognostic indicators for death. Arthritis Rheum.

[5] Tam LS, Tomlinson B, Chu TT, Li M, Leung YY, Kwok LW (2008). Cardiovascular risk profile of patients with psoriatic arthritis compared to controls–the role of inflammation. Rheumatology (Oxford).

[6] Tam LS, Kitas GD, Gonzalez-Gay MA (2014). Can suppression of inflammation by anti-TNF prevent progression of subclinical atherosclerosis in inflammatory arthritis?. Rheumatology (Oxford).

[7] Park S, Lakatta EG (2012). Role of inflammation in the pathogenesis of arterial stiffness. Yonsei Med J.

[8] Hansson GK (2005). Inflammation, atherosclerosis, and coronary artery disease. N Engl J Med.

[9] Eder L, Zisman D, Barzilai M, Laor A, Rahat M, Rozenbaum M (2008). Subclinical atherosclerosis in psoriatic arthritis: a case–control study. J Rheumatol.

[10] Eder L, Thavaneswaran A, Chandran V, Cook R, Gladman DD. Increased burden of inflammation over time is associated with the extent of atherosclerotic plaques in patients with psoriatic arthritis. Ann Rheum Dis. 2014. doi: 10.1136/annrheumdis-2014-205267. [Epub ahead of print] 10.1136/annrheumdis-2014-205267 24827532

[11] Giles JT, Post WS, Blumenthal RS, Polak J, Petri M, Gelber AC (2011). Longitudinal predictors of progression of carotid atherosclerosis in rheumatoid arthritis. Arthritis Rheum.

[12] Gonzalez-Gay MA, Gonzalez-Juanatey C, Pineiro A, Garcia-Porrua C, Testa A, Llorca J (2005). High-grade C-reactive protein elevation correlates with accelerated atherogenesis in patients with rheumatoid arthritis. J Rheumatol.

[13] Gonzalez-Gay MA, Gonzalez-Juanatey C, Lopez-Diaz MJ, Pineiro A, Garcia-Porrua C, Miranda-Filloy JA (2007). HLA-DRB1 and persistent chronic inflammation contribute to cardiovascular events and cardiovascular mortality in patients with rheumatoid arthritis. Arthritis Rheum.

[14] Cavalcante JL, Lima JA, Redheuil A, Al-Mallah MH (2011). Aortic stiffness: current understanding and future directions. J Am Coll Cardiol.

[15] Vlachopoulos C, Aznaouridis K, Terentes-Printzios D, Ioakeimidis N, Stefanadis C (2012). Prediction of cardiovascular events and all-cause mortality with brachial-ankle elasticity index: a systematic review and meta-analysis. Hypertension.

[16] Wu L, Wang Y, Zheng L, Li J, Hu D, Xu Y (2012). Distribution of brachial-ankle pulse wave velocity values and optimal cut-off in distinguishing subjects with clinical condition in Chinese population. Int Angiol.

[17] Costa L, Caso F, D’Elia L, Atteno M, Peluso R, Del Puente A (2012). Psoriatic arthritis is associated with increased arterial stiffness in the absence of known cardiovascular risk factors: a case control study. Clin Rheumatol.

[18] Soy M, Yildiz M, Sevki Uyanik M, Karaka N, Gufer G, Piskin S (2009). Susceptibility to atherosclerosis in patients with psoriasis and psoriatic arthritis as determined by carotid-femoral (aortic) pulse-wave velocity measurement. Rev Esp Cardiol (Engl Ed).

[19] Crilly MA, Kumar V, Clark HJ, Scott NW, Macdonald AG, Williams DJ (2009). Arterial stiffness and cumulative inflammatory burden in rheumatoid arthritis: a dose–response relationship independent of established cardiovascular risk factors. Rheumatology (Oxford).

[20] Maki-Petaja KM, Hall FC, Booth AD, Wallace SM, Yasmin, Bearcroft PW (2006). Rheumatoid arthritis is associated with increased aortic pulse-wave velocity, which is reduced by anti-tumor necrosis factor-alpha therapy. Circulation.

[21] Taylor W, Gladman D, Helliwell P, Marchesoni A, Mease P, Mielants H (2006). Classification criteria for psoriatic arthritis: development of new criteria from a large international study. Arthritis Rheum.

[22] Wong PC, Leung YY, Li EK, Tam LS (2012). Measuring disease activity in psoriatic arthritis. Int J Rheumatol.

[23] Fredriksson T, Pettersson U (1978). Severe psoriasis–oral therapy with a new retinoid. Dermatologica.

[24] Mease PJ. Measures of psoriatic arthritis: Tender and Swollen Joint Assessment, Psoriasis Area and Severity Index (PASI), Nail Psoriasis Severity Index (NAPSI), Modified Nail Psoriasis Severity Index (mNAPSI), Mander/Newcastle Enthesitis Index (MEI), Leeds Enthesitis Index (LEI), Spondyloarthritis Research Consortium of Canada (SPARCC), Maastricht Ankylosing Spondylitis Enthesis Score (MASES), Leeds Dactylitis Index (LDI), Patient Global for Psoriatic Arthritis, Dermatology Life Quality Index (DLQI), Psoriatic Arthritis Quality of Life (PsAQOL), Functional Assessment of Chronic Illness Therapy-Fatigue (FACIT-F), Psoriatic Arthritis Response Criteria (PsARC), Psoriatic Arthritis Joint Activity Index (PsAJAI), Disease Activity in Psoriatic Arthritis (DAPSA), and Composite Psoriatic Disease Activity Index (CPDAI). Arthritis Care Res (Hoboken). 2011;63:S64–85 10.1002/acr.20577 22588772

[25] Shang Q, Tam LS, Li EK, Yip GW, Yu CM (2008). Increased arterial stiffness correlated with disease activity in systemic lupus erythematosus. Lupus.

[26] Tam LS, Shang Q, Li EK, Wong S, Li RJ, Lee KL (2013). Serum soluble receptor for advanced glycation end products levels and aortic augmentation index in early rheumatoid arthritis–a prospective study. Semin Arthritis Rheum.

[27] Dessein PH, Joffe BI, Veller MG, Stevens BA, Tobias M, Reddi K (2005). Traditional and nontraditional cardiovascular risk factors are associated with atherosclerosis in rheumatoid arthritis. J Rheumatol.

[28] Shang Q, Tam LS, Sanderson JE, Sun JP, Li EK, Yu CM (2012). Increase in ventricular-arterial stiffness in patients with psoriatic arthritis. Rheumatology (Oxford).

[29] Ahlehoff O, Gislason GH, Charlot M, Jorgensen CH, Lindhardsen J, Olesen JB (2011). Psoriasis is associated with clinically significant cardiovascular risk: a Danish nationwide cohort study. J Intern Med.

[30] Ali Y, Tom BD, Schentag CT, Farewell VT, Gladman DD (2007). Improved survival in psoriatic arthritis with calendar time. Arthritis Rheum.

[31] Mok CC, Kwok CL, Ho LY, Chan PT, Yip SF (2011). Life expectancy, standardized mortality ratios, and causes of death in six rheumatic diseases in Hong Kong. China Arthritis Rheum.

[32] Wong K, Gladman DD, Husted J, Long JA, Farewell VT (1997). Mortality studies in psoriatic arthritis: results from a single outpatient clinic. I Causes and risk of death. Arthritis Rheum.

[33] Buckley C, Cavill C, Taylor G, Kay H, Waldron N, Korendowych E (2010). Mortality in psoriatic arthritis - a single-center study from the UK. J Rheumatol.

[34] Shbeeb M, Uramoto KM, Gibson LE, O’Fallon WM, Gabriel SE (2000). The epidemiology of psoriatic arthritis in Olmsted County, Minnesota, USA, 1982–1991. J Rheumatol.

[35] Gladman DD, Ang M, Su L, Tom BD, Schentag CT, Farewell VT (2009). Cardiovascular morbidity in psoriatic arthritis. Ann Rheum Dis.

[36] Westerweel PE, Verhaar MC (2009). Endothelial progenitor cell dysfunction in rheumatic disease. Nat Rev Rheumatol.

[37] Zieman SJ, Melenovsky V, Kass DA (2005). Mechanisms, pathophysiology, and therapy of arterial stiffness. Arterioscler Thromb Vasc Biol.

[38] Bailey AJ (2001). Molecular mechanisms of ageing in connective tissues. Mech Ageing Dev.

[39] Lee AT, Cerami A (1992). Role of glycation in aging. Ann NY Acad Sci.

[40] Throckmorton DC, Brogden AP, Min B, Rasmussen H, Kashgarian M (1995). PDGF and TGF-beta mediate collagen production by mesangial cells exposed to advanced glycosylation end products. Kidney Int.

[41] Yan SD, Schmidt AM, Anderson GM, Zhang J, Brett J, Zou YS (1994). Enhanced cellular oxidant stress by the interaction of advanced glycation end products with their receptors/binding proteins. J Biol Chem.

[42] Mease PJ (2002). Tumour necrosis factor (TNF) in psoriatic arthritis: pathophysiology and treatment with TNF inhibitors. Ann Rheum Dis.

[43] Gokce N, Vita JA, Loscalzo J, Schafer AI (2002). Clinical manifestations of endothelial dysfunction. Thrombosis and Hemorrhage.

[44] Angel K, Provan SA, Fagerhol MK, Mowinckel P, Kvien TK, Atar D (2012). Effect of 1-year anti-TNF-alpha therapy on aortic stiffness, carotid atherosclerosis, and calprotectin in inflammatory arthropathies: a controlled study. Am J Hypertens.

[45] Sugawara J, Hayashi K, Yokoi T, Cortez-Cooper MY, DeVan AE, Anton MA (2005). Brachial-ankle pulse wave velocity: an index of central arterial stiffness?. J Hum Hypertens.

